# Alpha fetoprotein (AFP) participates in the build up of hematopoietic cells in the early embryonic stage: an abortion case observation

**DOI:** 10.1186/s13000-019-0858-5

**Published:** 2019-08-01

**Authors:** Jianhong An, Yufeng Zhang, Jiao Luo, Hong Shen

**Affiliations:** 1 School of Medicine, South China University of Technology, Guangzhou Higher Education Mega Center, Guangzhou, 510006 Guangdong China; 2 Department of Pathology, School of Basic Medical Sciences, Southern Medical University, Guangzhou, 510515 Guangdong China; 3 Department of Gynecology, The Third Hospital of Nanhai, Foshan, 528200 Guangdong China; 4 Division of Nephrology, Nanfang Hospital, Southern Medical University, Guangzhou, 510515 Guangdong China

**Keywords:** Extramedullary hematopoiesis, Embryo, Blood island, Alpha fetoprotein

## Abstract

**Background:**

At the 3rd week of human embryo, some cell clumps are formed by the hyperplasia of mesenchymal cells at the germ layer of the yolk sac wall. These cell clumps are known as blood islands. The cells in the center of the blood islands further develop into primitive blood cells, such as hematopoietic stem cells. The blood island in the yolk sac further develops into the extramedullary hematopoietic tissue in 1 week at the 3rd to 4th week.

**Case presentation:**

A 32-year-old pregnant woman who missed menstruation for 42 days discovered that her pregnancy required an abortion. The tissue collected after the abortion was a piece of gray-yellow and villus-like intrauterine tissue of a size of approximately 4 cm × 3 cm × 1.3 cm. The paraffin section stained with hematoxylin and eosin and observed under the light microscope showed a visible small embryo tissue in the early placental tissue. In the embryonic tissue, a large amount of extramedullary hematopoietic tissue was present, including myeloid, erythroid and megakaryocytic cells. The extramedullary hematopoietic cells were located in the blood vessels or naive liver sinus, were positive for alpha fetoprotein (AFP) and were without lymphocytes. The erythrocytes consisted of a large number of nucleated red blood cells. In addition, a neural tube and cystic structure were found. The final pathological diagnosis was as follows: Early embryonic tissue with a cystic structure formation in the embryo. After medical abortion the pregnant woman recovered well, without complications.

**Conclusions:**

Our case illustrates that AFP is an important structural protein of nucleated erythrocytes and myeloid hematopoietic cells, suggesting that it may participate in the build up of nucleated erythrocytes and myeloid hematopoietic cells. Furthermore, our case suggests that nucleated red blood cells can be detected from the 42nd day of pregnancy by a peripheral blood sample from the mother.

## Background

At the 3rd week of human embryo, some cell clumps are formed by the hyperplasia of mesenchymal cells at the germ layer of yolk sac wall. These cell clumps are known as blood islands. The cells in the center of the blood islands further develop into primitive blood cells, such as hematopoietic stem cells. The blood island in the yolk sac further develops into the extramedullary hematopoietic tissue in 1 week at the 3rd to 4th week [1, 2]. Thus, the following questions arise associated to this phenomenon: is the blood island located inside or outside the blood vessels? What are the components of these extramedullary hematopoietic cells? Is the AFP related to the extramedullary hematopoietic cells early in the embryo? The answers for these questions have not been found in the literature [3–5].

## Case presentation

A 32-year-old pregnant woman was hospitalized after 42 days who missed menstruation and doctors found that her pregnancy required an abortion. The examination of color doppler ultrasonography showed an intrauterine early pregnancy with a pregnancy sac of 17 mm*18 mm*16 mm. The position of the pregnancy sac was biased towards the right corner and no germ was found. She asked for abortion. After admission, mifepristone and misoprostol were orally administered and the next morning the embryonic tissue was removed.

The gynecologic color ultrasonography showed the presence of a slightly strong echo area on the third day after the abortion, whose boundary was not defined, and the thickness of the echo area was approximately 13 mm in the uterine cavity. After the abortion, she recovered well, without complications.

## Materials and methods

The specimen was fixed in 10% formalin and embedded in paraffin. Hematoxylin and eosin (H&E) staining and immunohistochemistry were performed on 4 μm sections and observed under the light microscope. Immunostaining was performed using an autostainer (Leica Microsystems, Tokyo, Japan). Antibodies against the following antigens were used at the indicated dilutions: Platelet endothelial cell adhesion molecule-1 (CD31) (28364, Abcam; 1:50), Hematopoietic progenitor cell antigen CD34 (CD34) (81289, Abcam; 1:100), Hemoglobin (HB) (218019, Abcam; 1:200), Integrin beta-3 (CD61) (179475, Abcam; 1:250), myeloperoxidase (MPO) (136943, Abcam; 1:100), alpha fetoprotein (46799, Abcam; 1:200), S100 (14849, Abcam; 1:100), CD3 (11089, Abcam; 1:200), CD20 (78237, Abcam; 1:50), CD45RO (187836, Abcam; 1:200), and CD79a (187269, Abcam; 1:100).

## Results

### Pathological examination

**Fig. 1:**
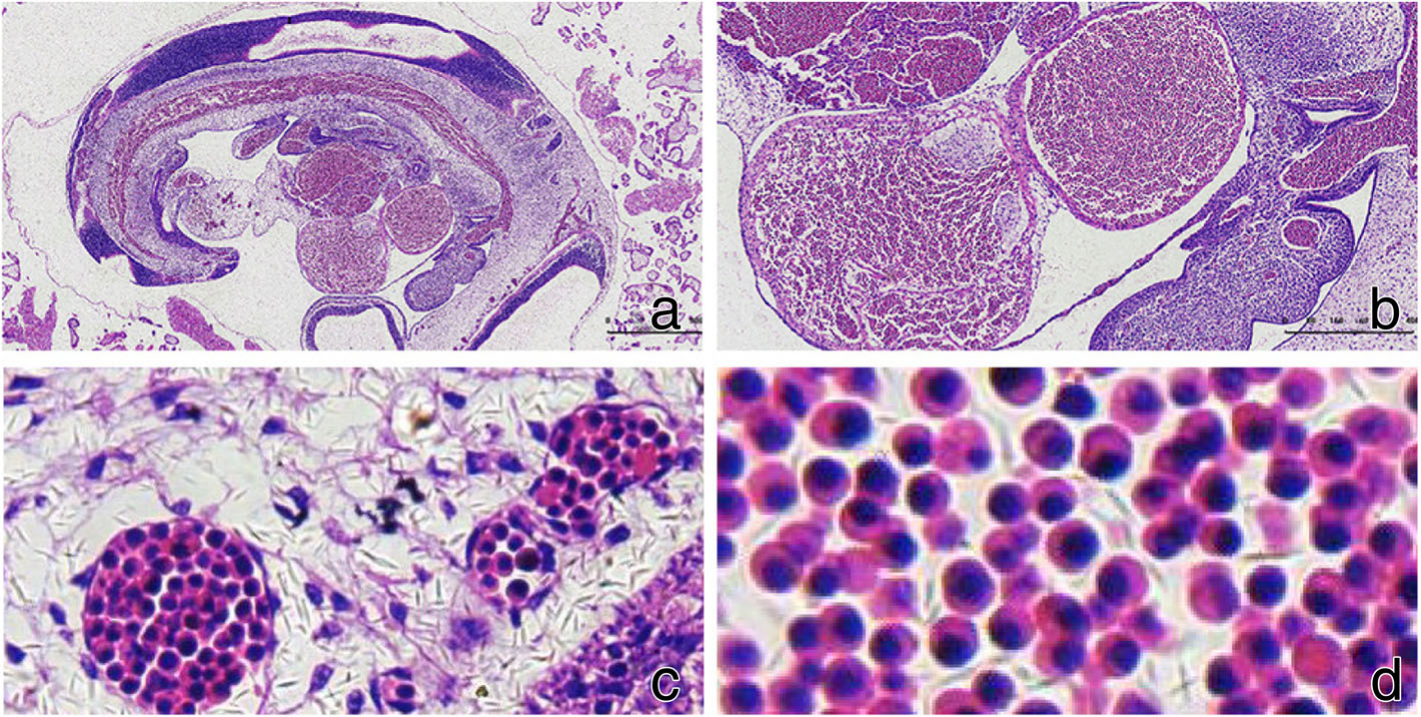
Early embryonic tissue and its nucleated erythrocytes (H&E staining). **a** shows the embryonic tissue (10× magnification), **b** shows embryonic extramedullary hematopoiesis (100× magnification), **c** shows intravascular extramedullary hematopoiesis (200× magnification), and **d** shows nucleated erythrocytes (400× magnification)

The tissue collected after the abortion was a piece of gray-yellow tissue with villi, with a size of approximately 4 cm*3 cm*1.3 cm. Light microscope observation revealed the following characteristics: a naive embryo and many early placental villi and trophoblast cells found in the tissue stained with H&E. Moreover, a large amount of extramedullary hematopoietic tissue was present in the embryo tissue. This extramedullary hematopoietic tissue was located in the embryo vessel, or in the liver sinus, and contained myeloid, erythroid and megakaryocytic cells. The erythroid cells consisted of a large number of nucleated erythrocytes, with a large and round shape and cytoplasm stained in red, color that was the same as the one of normal red blood cells (Fig. 1). In addition, a neural tube and a cystic structure were found.

### Immunohistochemical examination

**Fig. 2:**
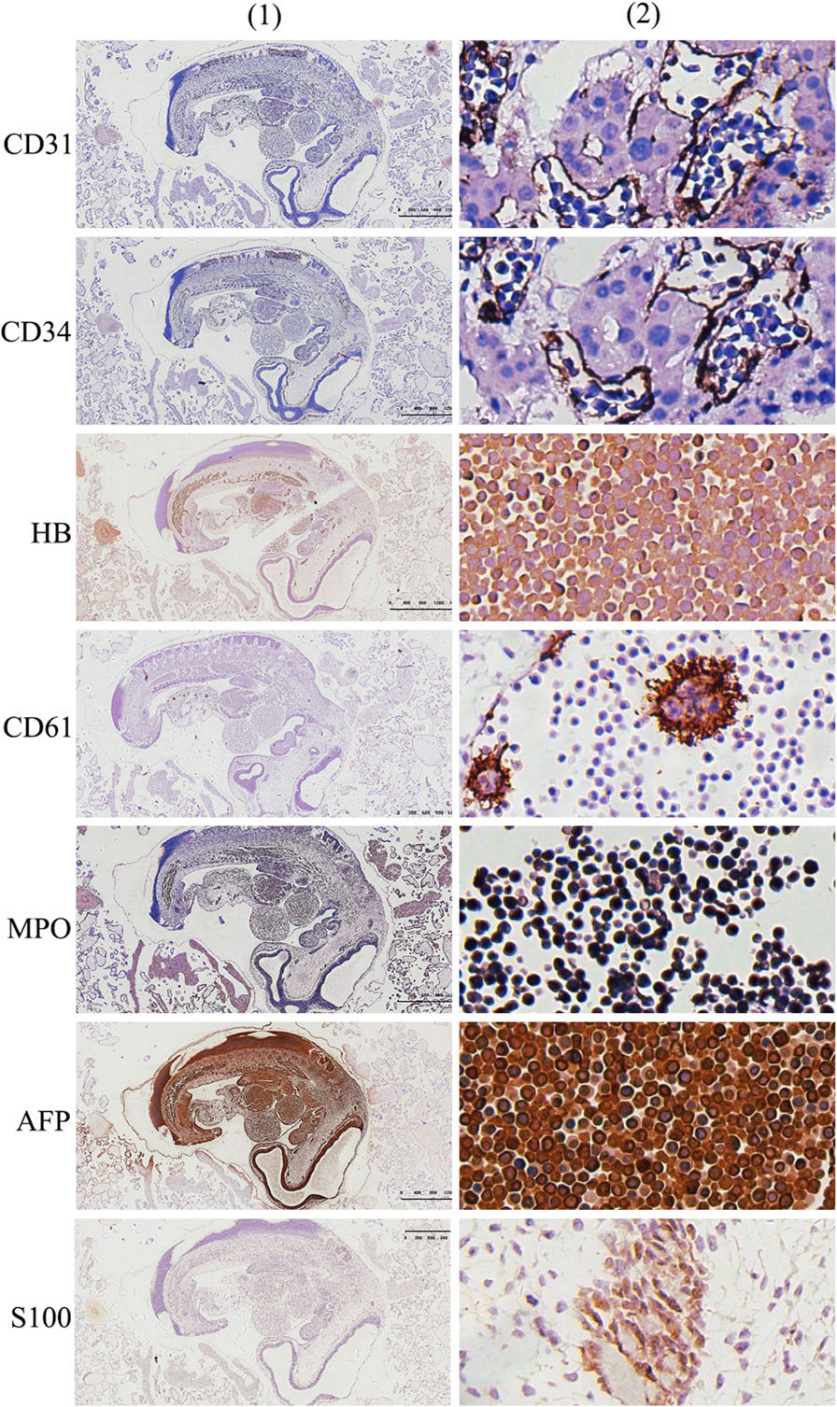
Immunohistochemical positive staining in the early embryonic tissue and its extramedullary hematopoietic tissue. CD31 positive staining in the vascular endothelial cells, suggesting that all the extramedullary hematopoietic cells were located in the blood vessels or naive liver sinus; CD34 positive staining in the vascular endothelial cells, suggesting that all the extramedullary hematopoietic cells were located in the blood vessels or naive liver sinus; CD61 positive staining in megakaryocytic cells, confirming that the positive cells are megakaryocyte; MPO positive staining in extramedullary hematopoietic celsl in the blood vessels, confirming that the positive cells are medullary hematopoietic cells; AFP positive staining in the extramedullary hematopoietic cells; S-100 positive staining in the neural tube; (1): 10× magnification; (2): 200× magnification)

**Fig. 3:**
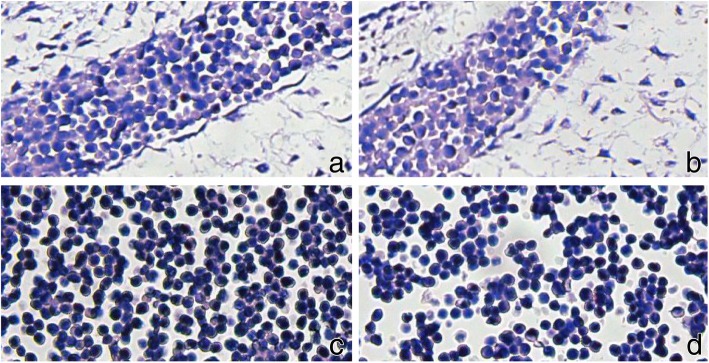
Immunohistochemical negative staining in early embryonic tissue and its extramedullary hematopoietic tissue. **a**, **b**, **c**, and **d** show the negative staining of CD3, CD20, CD45Ro, CD79a, respectively, suggesting that these cells are not lymphocytes. 200× magnification)

Positive staining was observed for CD31 (Vascular endothelial +, and all the extramedullary hematopoietic cells located in the blood vessels or naive liver sinus), CD34 (Vascular endothelial +, and all the extramedullary hematopoietic cells located in the blood vessels or naive liver sinus), HB (cytoplasm of nucleated erythrocytes +), MPO (extramedullary hematopoietic cell +), CD61 (megakaryocytic cell +), AFP (extramedullary hematopoietic cell +, including nucleated erythrocytes, myeloid cells and megakaryocytes), S100 (neural tube +), while CD3, CD45RO, CD20 and CD79a were negative. These results suggested that AFP is an important structural protein of nucleated erythrocytes and myeloid hematopoietic cells (Figs. 2 and 3).

### Pathological diagnosis

Early embryonic tissue with a cystic structure formation in the embryo.

## Discussion and conclusions

At the 3rd week of human embryo, some cell clumps are formed by the hyperplasia of mesenchymal cells at the germ layer of the yolk sac wall in the embryo. These cell clumps are known as blood islands. The cells in the center of the blood islands further develop into primitive blood cells, such as hematopoietic stem cells. The blood island in the yolk sac further develops into the extramedullary hematopoietic tissue in 1 week at the 3rd to 4th week [1, 2]. Thus, the following questions arise associated to this phenomenon: is the blood island located inside or outside the blood vessels? What are the components of these extramedullary hematopoietic cells? Is the AFP related to the extramedullary hematopoietic cells early in embryo? The answers for these questions have not been found in the literature [3–5].

Site of the extramedullary hematopoiesis in the early embryo and the composition of the extramedullary hematopoietic cells. The extramedullary hematopoietic cells and young blood vessels were observed in the embryo on the 4th week, but the bone marrow tissue was not formed, and the extramedullary hematopoietic cells were located in the blood vessels, based on the H&E staining and immunohistochemistry results. These extramedullary hematopoietic cells were composed of nucleated erythrocytes and myeloid hematopoietic cells, but no lymphocytes were present. These findings were consistent with those of other researchers, but our study illustrates these conclusions more vividly through different immunohistochemical stains. [6–9].

Relationship between AFP and the embryonic medullary hematopoiesis. It is reported that AFP is mainly synthesized in fetal liver and yolk sac, that it increases gradually after 13 weeks, and that is an important marker for fetal liver development [10]. In this case, AFP was also positive in nucleated erythrocytes and myeloid hematopoietic cells in the embryo on the 4th week, suggesting that AFP is also an important structural protein of nucleated erythrocytes and myeloid hematopoietic cells. In addition, it also suggests that AFP may participate in the build up of nucleated erythrocytes and myeloid hematopoietic cells.

Clinical significance of nucleated erythrocytes. The fetal nucleated erythrocyte contains a complete nucleus, thus, it carries an intact fetal gene set and can persist in maternal blood during pregnancy. The peripheral blood of a woman who is not pregnant does not contain nucleated red blood cells; therefore, the fetal nucleated red blood cells in the mother’s blood can be used as a fetal non-invasive prenatal diagnosis of hereditary diseases [11]. It is generally believed that nucleated red blood cells can be detected in the peripheral blood of pregnant women at 7~34 weeks of pregnancy [12]. This specific case revealed that nucleated red blood cells could be used from the 42nd day of pregnancy by a peripheral blood sample from the mother.

Significance of the immunohistochemical staining results.

1. CD31 is a platelet-endothelial cell adhesion molecule. In this case, the vascular endothelial expression was positive, indicating that the original blood vessel was formed at the beginning of the 4th week (28th day) of the embryo. Both CD31 and CD34 can be used to stain endothelial cells and subsequently blood vessels. Thanks to the vascular markers of CD31 and CD34, it was clear that the extramedullary hematopoietic cells were located only in the blood vessels, indicating that the extramedullary hematopoiesis was initially performed in the blood vessels.
2. HB is a hemoglobin marker, which can be used to stain the cytoplasm of nucleated erythrocytes. The positive staining in this case proved that these cells were nucleated erythrocytes in the extramedullary hematopoietic cells.
3. MPO is a marker of myeloid hematopoietic cells [13]. The positive staining with a large number of positive cells in this case indicated the presence of many myeloid hematopoietic cells in the extramedullary hematopoietic cells in the blood vessel.
4. CD61 is a platelet-associated antigen, which is a marker of megakaryocytic cell. In this sample, the CD61 positive staining indicated that the giant nuclear cells of medullary hematopoietic cells in the vessel were megakaryocytic cells, and implied that platelets could be produced at least starting from the 4th week in the embryo.
5. Both CD3 and CD45Ro are T-lymphocyte markers, both CD20 and CD79a are B-lymphocyte markers, and CD34 can also mark immature B lymphocytes. These 5 markers were negative in the sample from our patient, suggesting the absence of lymphocytes in the extramedullary hematopoietic cells and in the embryo at least in the 4th week.
6. AFP was positive in the extramedullary hematopoietic cells including nucleated erythrocytes, myeloid hematopoietic cells and megakaryocytic cells, suggesting that AFP may play an important role in the development of extramedullary hematopoietic cells in early embryos.
7. S-100 marker can be used to observe the neural tube structures in tissues [14].

In conclusion, this paper reports a case of 4-week-old embryo from an abortion, which shows that early embryonic extramedullary hematopoiesis takes place in the young blood vessels and that the fetal extramedullary hematopoietic system develops earlier, at least at the beginning of the 4th week. The formation of erythrocytes, myeloid hematopoietic cells and megakaryocytic cells is earlier than that of lymphoid cells. In addition, AFP appears to be an important structural protein of the myeloid hematopoietic cells, erythrocytes and megakaryocytic cell during the early development of embryo, which participates in the build up of hematopoietic cells in the early embryonic stage. This case also suggests that the maternal peripheral blood during pregnancy can be used for noninvasive prenatal genetic examination based on the nucleated erythrocytes, examination can begin from the 42 days of pregnancy.

## Abbreviations

AFP: alpha fetoprotein
H&E: hematoxylin and eosin
HB: hemoglobin
MPO: myeloperoxidase
